# Mucin1 promotes the migration and invasion of hepatocellular carcinoma cells via JNK-mediated phosphorylation of Smad2 at the C-terminal and linker regions

**DOI:** 10.18632/oncotarget.4267

**Published:** 2015-05-25

**Authors:** Juan Wang, Guomu Liu, Qiongshu Li, Fang Wang, Fei Xie, Ruiping Zhai, Yingying Guo, Tanxiu Chen, Nannan Zhang, Weihua Ni, Hongyan Yuan, Guixiang Tai

**Affiliations:** ^1^ Department of Immunology, College of Basic Medical Science, Jilin University, Changchun, China

**Keywords:** Mucin1, HCC, JNK, TGF-β, Smad2L/C

## Abstract

Mucin1 (MUC1), as an oncogene, plays a key role in the progression and tumorigenesis of many human adenocarcinomas. In this study, wound-healing, transwell migration and matrigel invasion assays showed that MUC1 promotes human hepatocellular carcinoma (HCC) cell migration and invasion by MUC1 gene silencing and overexpressing. Treatment with exogenous transforming growth factor beta (TGF-β)1, TGF-β type I receptor (TβRI) inhibitor, TGF-β1 siRNAs, or activator protein 1 (AP-1) inhibitor to MUC1-overexpressing HCC cells revealed that MUC1-induced autocrine TGF-β via JNK/AP-1 pathway promotes the cell migration and invasion. In addition, the migration and invasion of HCC cells were more significantly inhibited by JNK inhibitor compared with that by TβRI inhibitor or TGF-β1 siRNAs. Further studies demonstrated that MUC1-mediated JNK activation not only enhances the phosphorylation of Smad2 C-terminal at Ser-465/467 site (Smad2C) through TGF-β/TβRI, but also directly enhances the phosphorylation of Smad2 linker region at Ser-245/250/255 site (Smad2L), and then both of them collaborate to upregulate matrix metalloproteinase (MMP)-9-mediated cell migration and invasion of HCC. These results indicate that MUC1 is an attractive target in liver cancer therapy.

## INTRODUCTION

Mucin1 (MUC1) is a transmembrane glycoprotein that is expressed on the apical surface of epithelial cells and is aberrantly overexpressed on most epithelial malignant tumors and some hematological malignant tumors, and it promotes the progression and tumorigenesis of many human adenocarcinomas [1-5]. MUC1 consists of a large extracellular N-terminal subunit and a C-terminal subunit that reside on the cell surface as a heterodimeric complex via strong noncovalent interactions [6]. The C-terminal subunit is composed of a 58-amino acid extracellular domain, a 28-amino acid transmembrane domain (TM), and a 72-amino acid cytoplasmic tail (CT) [6]. MUC1-CT is involved in many signaling pathways, including Wnt/β-catenin [7], c-terminal Src kinase (c-Src) [8], growth factor receptor-bound protein 2 (Grb2)/son of sevenless (Sos) [9], phosphatidylinositol-3-kinase (PI3K)/protein kinase B (AKT) [2], p53 [10], glycogen synthase kinase 3β (GSK3β) [6], epidermal growth factor receptor (EGFR) [11, 12], nuclear factor-κB (NF-κB) [13], and c-Jun N-terminal kinase (JNK) [14], to regulate the processes of cell survival, proliferation, and apoptosis. There is additional evidence that MUC1 contributes to the cell migration, invasion, and metastasis properties of tumors via several proteins, such as intercellular adhesion molecule 1 (ICAM-1), E-cadherin, Wnt/β-catenin, c-Src, c-Met and γ-secretase [15-20].

Human hepatomacellular carcinoma (HCC) is one of the most common malignant tumors and has severely threatened human health and quality of life worldwide. Various reports and our previous studies have shown that MUC1 is overexpressed in HCC cells and tissues [21, 22]. Our previous results revealed that MUC1 gene silencing inhibited the growth of the SMMC-7721 HCC cell line *in vivo* and *in vitro*, suggesting that MUC1 plays a key role in HCC tumorigenesis. However, whether MUC1 could promote the migration and invasion of HCC cells remains unclear. In a recent study, we showed that MUC1 induced autocrine transforming growth factor beta (TGF-β) in HCC cells [23], leading to the hypothesis that MUC1 might activate TGF-β signaling to promote the migration and invasion of HCC cells.

TGF-β, a multifunctional cytokine, plays a tumor suppressive role in normal epithelia cells and precancerous tissues by inhibiting cell proliferation and inducing apoptosis, but accelerates the progression of established cancers by promoting cell proliferation, invasion, and metastasis [24-27]. TGF-β initiates signaling by binding to type I and type II receptor serine/threonine kinases on the cell surface, receptor-mediated Smads activation to regulate gene expression, which is a classical pathway for TGF-β signaling transduced from the cell membrane to the nucleus, and resulting in tumor suppression. Moreover, TGF-β also promotes the progression of tumors by the activation of various oncogene signalings via Smad-independent signaling pathway [28, 29]. Smads, central mediators converting TGF-β signaling from receptors to the nucleus, consist of three domains: Mad homology (MH)1, intermediate linker, and MH2 domains. The catalytically active TGF-β type I receptor (TβRI) could phosphorylate Smad2 at the C-terminal Ser-465/467 site (Smad2C) [30], while JNK could phosphorylate Smad2 at the linker region Ser-245/250/255 site (Smad2L) [31]. Furthermore, activated TβRI and JNK together could create cytoplasmic the phosphorylation of both Smad2L/C, which entered the nucleus by rapidly oligomerizing with Smad4, promoting cell migration and invasion partly by upregulating PAI-1, matrix metalloproteinase (MMP)-1, MMP-2, and MMP-9 in mice ﬁbroblasts and human colorectal cancers [32, 33]. These results reveal that JNK-induced activation of Smad2 in the TGF-β signal promotes the migration and invasion of HCC cells. However, the JNK activation mechanism is still unclear. A recent study has showed that MUC1 can activate JNK1 to inhibit cisplatin-induced apoptosis in human colon cancer HCT116 cells [14]. Our latest study also has shown that MUC1-induced activation of JNK enhances the proliferation by mediating Smad3 signaling [34]. Thus, leading to the hypothesis that MUC1 could activate Smad2 in TGF-β signaling by activating JNK to promote the migration and invasion of HCC cells.

In this study, we use MUC1 gene-silenced and overexpressing HCC cells to determine if MUC1 can promote the migration and invasion of HCC cells and clarify whether TGF-β/Smad2 and JNK are involved in the mechanisms that MUC1 promotes the migration and invasion of HCC cells, providing a novel therapeutic target for the pathogenesis and gene therapy of HCC.

## RESULTS

### MUC1 promotes the migration and invasion of HCC cells

**Figure 1:**
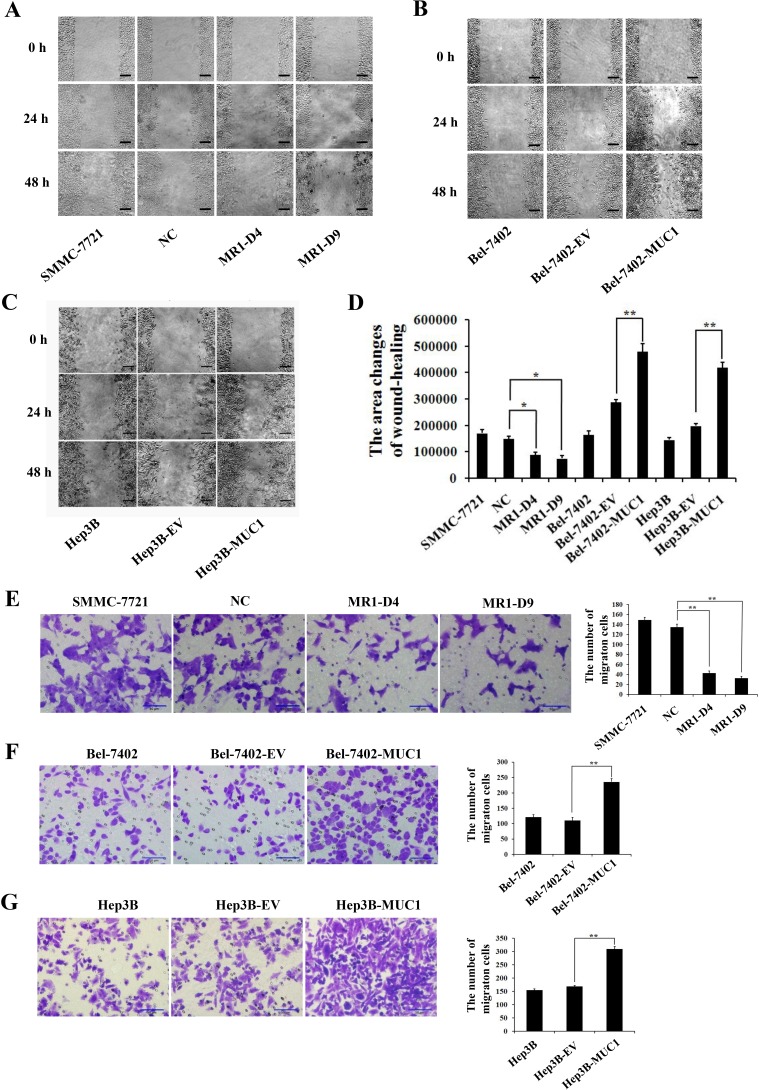
MUC1 promotes the migration of HCC cells **A.**−**C.** The migration of MUC1-knockdown SMMC-7721 cells **A.**, and MUC1-overexpressing Bel-7402 **B.** and Hep3B **C.** cells were detected by wound-healing assay. The photographs were taken by microscope (IX71; OLYMPUS) using a 100 × magnification at the same area at 0 h and after 24 h and 48 h of incubation of five random fields. The scale bar indicates 100 μm. **D.** The wound-healing assay was assessed by measuring the pixels of the wound-healing area using Image-Pro Plus 6.0 software. Bars represent the changes in wound-healing area within 48 h. **E.**−**G.** The migration of MUC1-knockdown SMMC-7721 cells **E.**, and MUC1-overexpressing Bel-7402 **F.** and Hep3B **G.** cells were detected by transwell migration assay. Migrated cells were counted in five random fields of each filter under a microscope (IX71; OLYMPUS) using a 200 × magnification. The scale bar indicates 50 μm. Bars represent the average number of migrated cells. MR1-D4 and MR1-D9, MUC1-knockdown cells; NC, the negative control of MUC1-knockdown cells; Bel-7402-MUC1 and Hep3B-MUC1, MUC1-overexpressing cells; Bel-7402-EV and Hep3B-EV, the negative controls of Bel-7402-MUC1 and Hep3B-MUC1, respectively. *P < 0.05, **P < 0.01 compared with respective controls.

**Figure 2:**
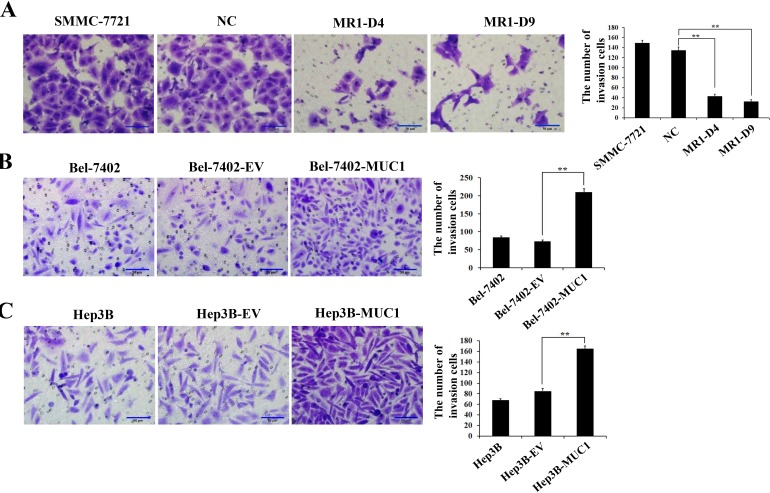
MUC1 promotes the invasion of HCC cells **A.**−**C.** The invasion of MUC1-knockdown SMMC-7721 cells **A.**, and MUC1-overexpressing Bel-7402 **B.** and Hep3B **C.** cells were detected by matrigel invasion assay. Cells that invaded across the matrigel of the transwell were counted in five random fields of each filter under a microscope (IX71; OLYMPUS) using a 200 × magnification. The scale bar indicates 50 μm. Bars represent the average number of invaded cells. **P < 0.01 compared with respective controls.

To determine the effect of MUC1 on the migration and invasion of HCC cells, wound-healing, transwell migration, and matrigel invasion assays were performed using two independent MUC1-knockdown clones (MR1-D4 and MR1-D9) and two MUC1-overexpressing cell lines (Bel-7402-MUC1 and Hep3B-MUC1) that were previously established [22, 23]. In the wound-healing assay, the results showed that the area changes for wound-healing in the MR1-D4 and MR1-D9 cells were reduced compared with the SMMC-7721 or the NC cells (*P* < 0.05) (Figure 1A and 1D). In the Bel-7402-MUC1 and the Hep3B-MUC1 cells, the area changes of wound-healing were significantly increased compared with the respective controls (*P* < 0.01) (Figure 1B–1D). In transwell migration and matrigel invasion assays, the results showed that the cells in the lower chamber of transwell were obviously decreased in MUC1-knockdown cells, compared with SMMC-7721 or NC (*P* < 0.01) (Figures 1E and 2A); in contrast, the cells in the lower chamber of transwell were significantly increased in MUC1-overexpressing cells compared with the control, respectively (*P* < 0.01) (Figures 1F, 1G and 2B, 2C). Taken together, these results indicate that MUC1 promotes both the migration and invasion of HCC cells.

### MUC1-induced TGF-β promotes the migration and invasion of HCC cells

**Figure 3:**
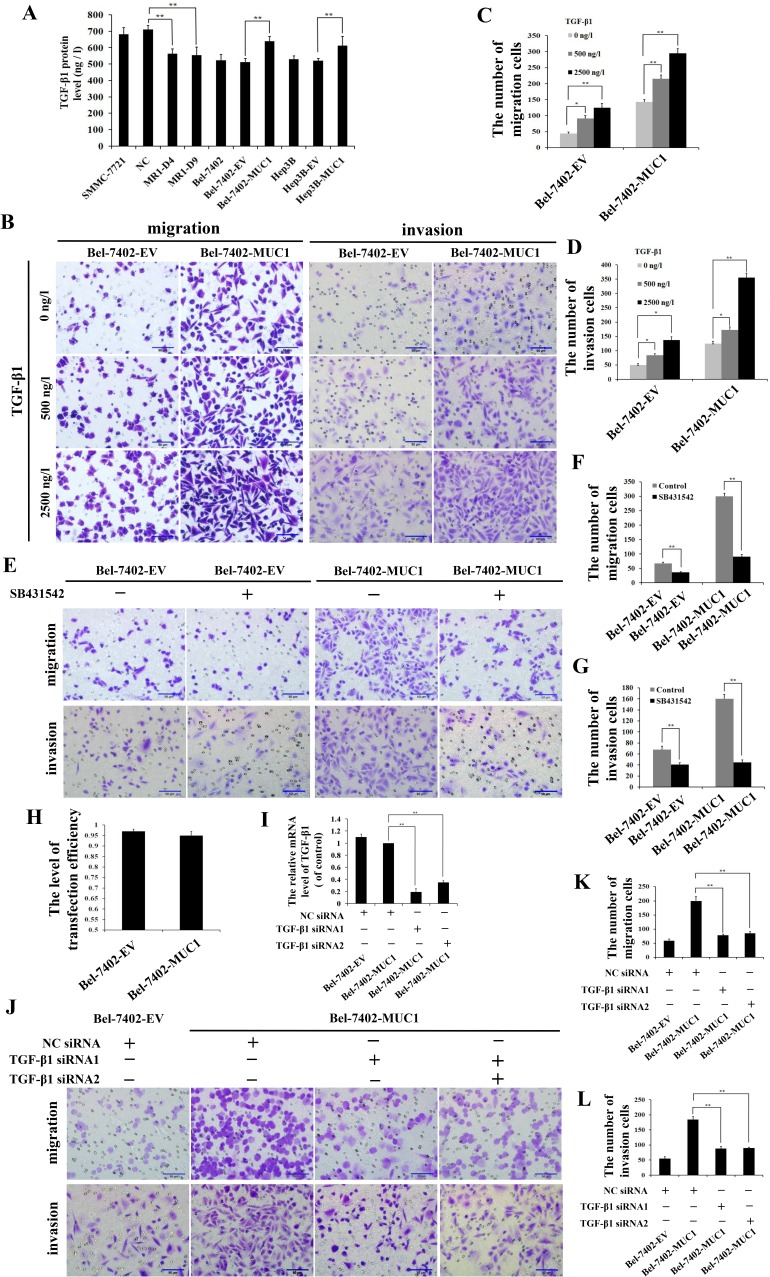
MUC1-induced TGF-β promotes the migration and invasion of HCC cells **A.** TGF-β1 levels in the cell culture supernatants of MUC1-knockdown and MUC1-overexpressing cells were measured by ELISA. Bars represent the TGF-β protein levels of those cells. **B.**−**G.** The effect of different doses of exogenous TGF-β1 **B.**−**D.** or SB431542 (30 μM) **E.**−**G.** on the migration and invasion of Bel-7402-EV and Bel-7402-MUC1 cells were detected by transwell migration and matrigel invasion assays, respectively. Migrated or invaded cells were counted in five random fields of each filter under a microscope (IX71; OLYMPUS) using a 200 × magnification. The scale bar indicates 50 μm. Bars represent the average number of migrated or invaded cells. **H.** The transfection efficiency levels of Bel-7402-EV and Bel-7402-MUC1 cells were detected by NC siRNA-Cy3. Bars represent the transfection efficiency levels when transfected with TGF-β1 siRNAs. **I.** Bel-7402-EV and Bel-7402-MUC1 cells were transfected with NC-siRNA or TGF-β siRNAs, and then the mRNA of TGF-β1 was detected by qRT-PCR. Bars represent the relative mRNA level of TGF-β1 when compared to Bel-7402-MUC1-NC siRNA group. **J.**−**L.** Bel-7402-EV and Bel-7402-MUC1 cells were transfected with NC siRNA or TGF-β1 siRNAs, and then the migration and invasion of those cells were detected by transwell migration and matrigel invasion assays, respectively. Cells that migrated or invaded across the membrane of the transwell were counted in five random fields of each filter under a microscope (IX71; OLYMPUS) using a 200 × magnification. The scale bar indicates 50 μm. Bars represent the average number of migrated or invaded cells. The data are expressed as the mean ± SD of three independent experiments. *P < 0.05, **P < 0.01 compared with respective controls.

To study the mechanism of MUC1-enhanced HCC cell migration and invasion, autocrine TGF-β1 levels in both MUC1-knockdown and overexpressing HCC cells were detected by ELISA. The results showed that the autocrine TGF-β1 was inhibited in the MUC1-knockdown cells (MR1-D4 and MR1-D9), while the TGF-β1 levels in MUC1-overexpressing cells (Bel-7402-MUC1 and Hep3B-MUC1) were increased significantly compared with the control groups (*P* < 0.01), and approximately 600−700 ng/l of the autocrine TGF-β1 in MUC1-overexpressing cells was produced (Figure 3A). These results further confirm that MUC1 enhances the autocrine TGF-β in HCC cells. Subsequently, to detect the effect of MUC1-induced TGF-β on cell migration and invasion, different doses of exogenous TGF-β1 were added to the culture media of Bel-7402-EV and Bel-7402-MUC1 HCC cells. The results showed that Bel-7402-MUC1 cells were more migratory and invasive than Bel-7402-EV cells in the presence of the same concentration of exogenous TGF-β1 (Figure 3B–3D). To further verify the effect of the autocrine TGF-β on cell migration and invasion, SB431542 (30 μM), an inhibitor of TβRI, was used to block the TGF-β/TβRI pathway. The results showed that SB431542 inhibited the migration and invasion of both Bel-7402-MUC1 and Bel-7402-EV cells, and the inhibitory effect on Bel-7402-MUC1 cells was greater than that on Bel-7402-EV cells (Figure 3E–3G). Furthermore, Bel-7402-MUC1 cells were transfected with two siRNAs targeting TGF-β1 using Lipofectamine 2000. Figure 3H shows that the transfection efficiency of siRNAs reached 95% and the silencing efficiency of the TGF-β gene induced by TGF-β1 siRNA1 and TGF-β1 siRNA2 reached approximately 80.35% and 65.83%, respectively (Figure 3I). The migration and invasion of Bel-7402-MUC1 cells were markedly inhibited by both TGF-β1 siRNA1 and TGF-β1 siRNA2, compared with NC siRNA (*P* < 0.01) (Figure 3J–3L). These results suggest that MUC1-induced TGF-β upregulates HCC cell migration and invasion.

### MUC1-induced autocrine TGF-β through activation of JNK promotes the migration and invasion of HCC cells

**Figure 4:**
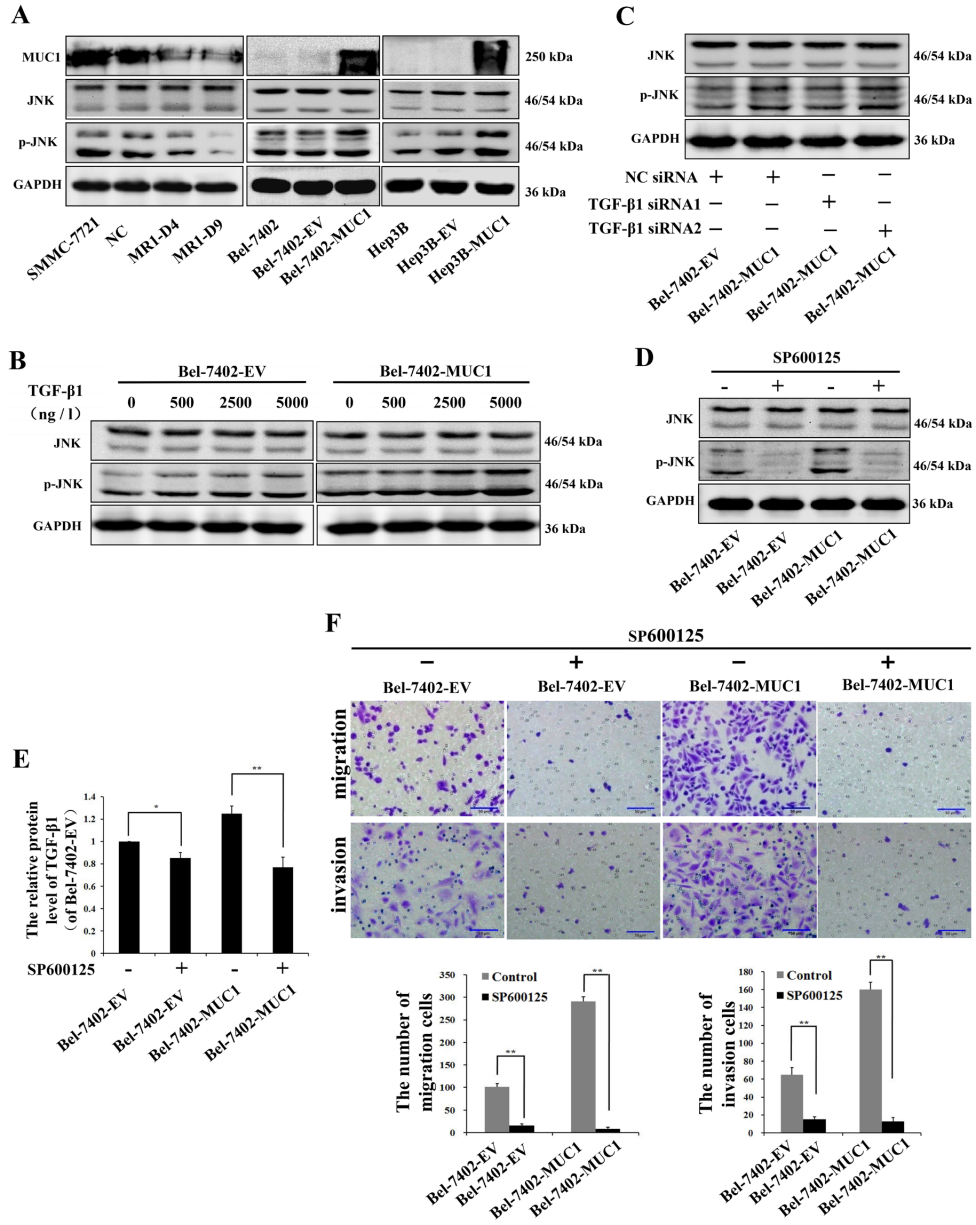
MUC1-induced phosphorylation of JNK promotes the migration and invasion of HCC cells **A.** Cell lysates of MUC1-knockdown cells, and MUC1-overexpressing cells were analyzed by Western blotting for the levels of MUC1, JNK and p-JNK; GAPDH was used as a loading control. **B.**−**D.** The effect of different doses of exogenous TGF-β1 **B.**, NC siRNA or TGF-β1 siRNAs **C.**, and JNK inhibitor SP600125 (30 μM) **D.** on the expression of JNK and p-JNK in Bel-7402-EV and Bel-7402-MUC1 cells were analyzed by Western blotting; GAPDH was used as a loading control. **E.** The expression of TGF-β1 in Bel-7402-EV and Bel-7402-MUC1 cells when treated with or without SP600125 (30 μM) were assayed by ELISA. Bars represent the relative protein levels of TGF-β1 when compared to Bel-7402-EV (SP600125−). The data are expressed as the mean ± SD of three independent experiments. **F.** The migration and invasion of Bel-7402-EV and Bel-7402-MUC1 cells when treated with or without SP600125 (30 μM) were detected by transwell migration and matrigel invasion assays, respectively. Migrated or invaded cells were counted in five random fields of each filter under a microscope (IX71; OLYMPUS) using a 200 × magnification. The scale bar indicates 50 μm. Bars represent the average number of migrated or invaded cells. *P < 0.05, **P < 0.01 compared with respective controls.

We found that the effect of MUC1 upregulating HCC cell migration and invasion is correlated to MUC1-induced TGF-β, but the mechanisms remained largely unknown. Our previous study had shown that MUC1 facilitated the autocrine TGF-β via the JNK/AP-1 pathway in HCC cells [23]. Therefore, we speculated that MUC1-induced activation of JNK enhances the autocrine TGF-β, which could promote the subsequent migration and invasion of HCC cells. To test this, Western blotting analysis was performed, and the results showed that the phosphorylation of JNK was significantly elevated in the MUC1-overexpressing cell lines (Bel-7402-MUC1 and Hep3B-MUC1) compared with the respective control cells. In contrast, knockdown of MUC1 in SMMC-7721 cells (MR1-D4 and MR1-D9) inhibited JNK activation (Figure 4A). These results suggested that MUC1 enhances the phosphorylation of JNK. As several other studies have shown that TGF-β could activate JNK by mediating Smad-independent signaling [28, 29], to further examine the effect of MUC1-induced TGF-β on the phosphorylation of JNK, we treated cells with different doses of exogenous TGF-β1 or TGF-β1 siRNAs, and the phosphorylation of JNK were detected by Western blotting. The results showed that in the presence of 2500 ng/l and 5000 ng/l exogenous TGF-β1, JNK was obviously activated both in Bel-7402-EV and Bel-7402-MUC1 cells, but in the presence of 500 ng/l exogenous TGF-β1, which was similar to the concentration of the autocrine TGF-β1 (Figure 3A) or TGF-β1 siRNAs, there were no significant differences in the activation of JNK both in MUC1-overexpressing cells and the respective control cells (Figure 4B and 4C). Taken together, these results indicate that JNK activation is enhanced by MUC1 but not MUC1-induced TGF-β in HCC cells. To further clarify that the migration and invasion were promoted by the MUC1/JNK/TGF-β signaling pathway in HCC cells, JNK was blocked by the specific inhibitor SP600125 (30 μM), and the mRNA of TGF-β1 were detected by qRT-PCR. The results showed that the autocrine TGF-β1 was significant downregulated by 38.27% when the activation of JNK was inhibited by SP600125 in Bel-7402-MUC1 cells (Figure 4D and 4E). The migration and invasion of both MUC1-overexpressing cells (Bel-7402-MUC1) and the control cells were almost abolished by SP600125 (*P* < 0.01) (Figure 4F). In addition, TGF-β signaling was blocked by SB431542 and TGF-β1 siRNAs, which was also performed as described in Figure 3. The results showed that the amount of Bel-7402-MUC1 cell migration and invasion were reduced by blocking TGF-β signaling was less than that caused by blocking JNK signaling with SP600125. These results revealed that MUC1-enhanced activation of JNK promoted the migration and invasion of HCC cells partly through TGF-β signal.

### MUC1 through JNK-mediated phosphorylation of Smad2L/C promotes the migration and invasion of HCC cells

**Figure 5:**
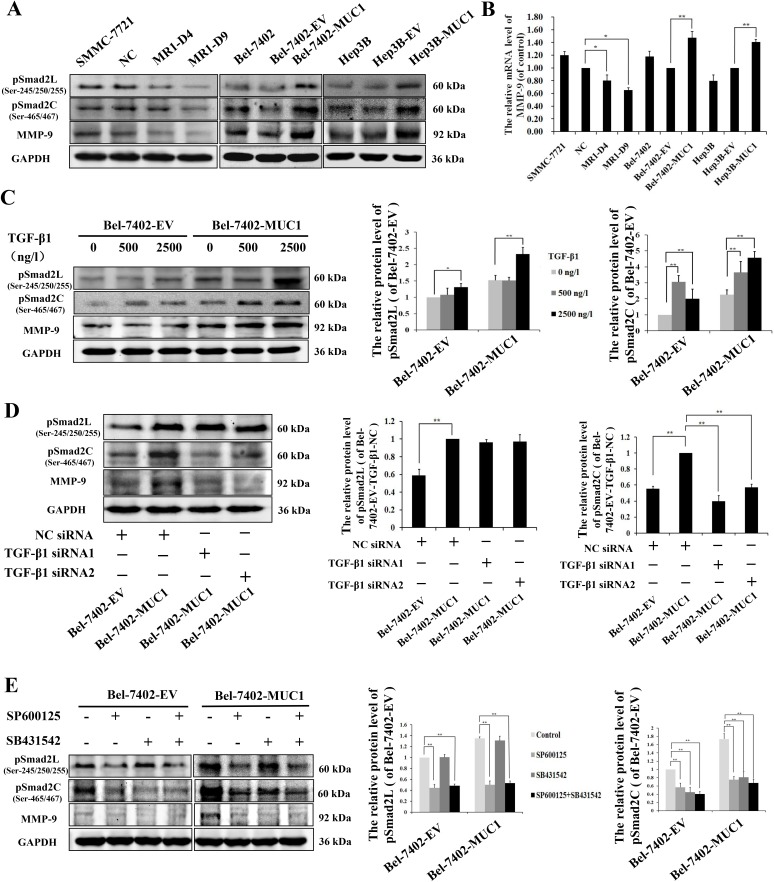
MUC1 through JNK-mediated phosphorylation of Smad2L/C promotes the MMP-9-mediated cell migration and invasion of HCC **A.** Cell lysates of MUC1-knockdown SMMC-7721 cells, and MUC1-overexpressing Bel-7402 and Hep3B cells were analyzed by Western blotting for the phosphorylation of Smad2L (pSmad2L) (Ser-245/250/255), the phosphorylation of Smad2C (pSmad2C) (Ser-465/467), and MMP-9; GAPDH was used as a loading control. **B.** The mRNA of MMP-9 in MUC1-knockdown SMMC-7721 cells, and MUC1-overexpressing Bel-7402 and Hep3B cells were detected by qRT-PCR. Bars represent the relative mRNA level of MMP-9 when compared to the respective controls. **C.**−**E.** The effect of different doses of exogenous TGF-β1 **C.**, NC siRNA or TGF-β1 siRNAs **D.**, and JNK inhibitor SP600125 (30 μM) or TβRI inhibitor SB431542 (20 μM) **E.** on the levels of pSmad2L, pSmad2C, and MMP-9 in Bel-7402-EV and Bel-7402-MUC1 cells were analyzed by Western blotting; GAPDH was used as a loading control. Bars represent the relative protein levels of pSmad2L and pSmad2C when compared to the respective controls. The data are expressed as the mean ± SD of three independent experiments. *P < 0.05, **P < 0.01 compared with respective controls.

To further investigate the molecular mechanisms of MUC1/JNK/TGF-β upregulated migration and invasion in HCC cells, the phosphorylation of Smad2L/C in TGF-β signaling pathway and the expression of its target gene MMP-9 were detected by Western blotting and/or qRT-PCR in both MUC1 gene silenced and overexpressing HCC cells. The results showed that knockdown of MUC1 downregulated the phosphorylation of Smad2L/C and the expression of MMP-9, while in MUC1-overexpressing cells, the phosphorylation of Smad2L/C and the expression of MMP-9 were significantly upregulated compared with the control (Figure 5A and 5B).

Subsequently, to clarify whether Smad2C were phosphorylated by activated TβRI, Bel-7402-EV and Bel-7402-MUC1 cells were stimulated with different doses of exogenous TGF-β1. Western blotting analysis showed that in Bel-7402-MUC1 cells, a low concentration of exogenous TGF-β1 (500 ng/l), which was similar to the concentration of the autocrine TGF-β1 (Figure 3A), had no effect on the phosphorylation of Smad2L but that a higher concentration, 2500 ng/l, for example, did have an effect, whereas TGF-β1 had a dose-dependent effect on the phosphorylation of Smad2C. The expression of MMP-9 was increased significantly in Bel-7402-MUC1 cells than that of Bel-7402-EV cells (Figure 5C). Furthermore, when TGF-β/TβRI was blocked by TGF-β1 siRNAs in Bel-7402-MUC1 cells, the phosphorylation of Smad2C and the expression of MMP-9 was decreased, but the phosphorylation of Smad2L was not affected (Figure 5D). These results demonstrated that TGF-β/TβRI enhanced the phosphorylation of Smad2C. To further determine the mechanism of how Smad2L/C was phosphorylated, JNK inhibitor SP600125 (30 μM) and TβRI inhibitor SB431542 (30 μM) were applied individually or in combination in both Bel-7402-EV and Bel-7402-MUC1 cells. Western blotting analysis showed that SP600125 inhibited the phosphorylation of Smad2L/C and the expression of MMP-9, while SB431542 inhibited the phosphorylation of Smad2C and the expression of MMP-9 but not the phosphorylation of Smad2L (Figure 5E). Taken together, these results demonstrate that MUC1-induced JNK activation not only enhances the phosphorylation of Smad2C through TGF-β/TβRI, but also directly enhances the phosphorylation of Smad2L, and then both of them collaborate to upregulate the MMP-9-mediated cell migration and invasion of HCC cells.

### MUC1 promotes the migration and invasion of HCC cells through the JNK/AP-1/TGF-β signaling pathway

**Figure 6:**
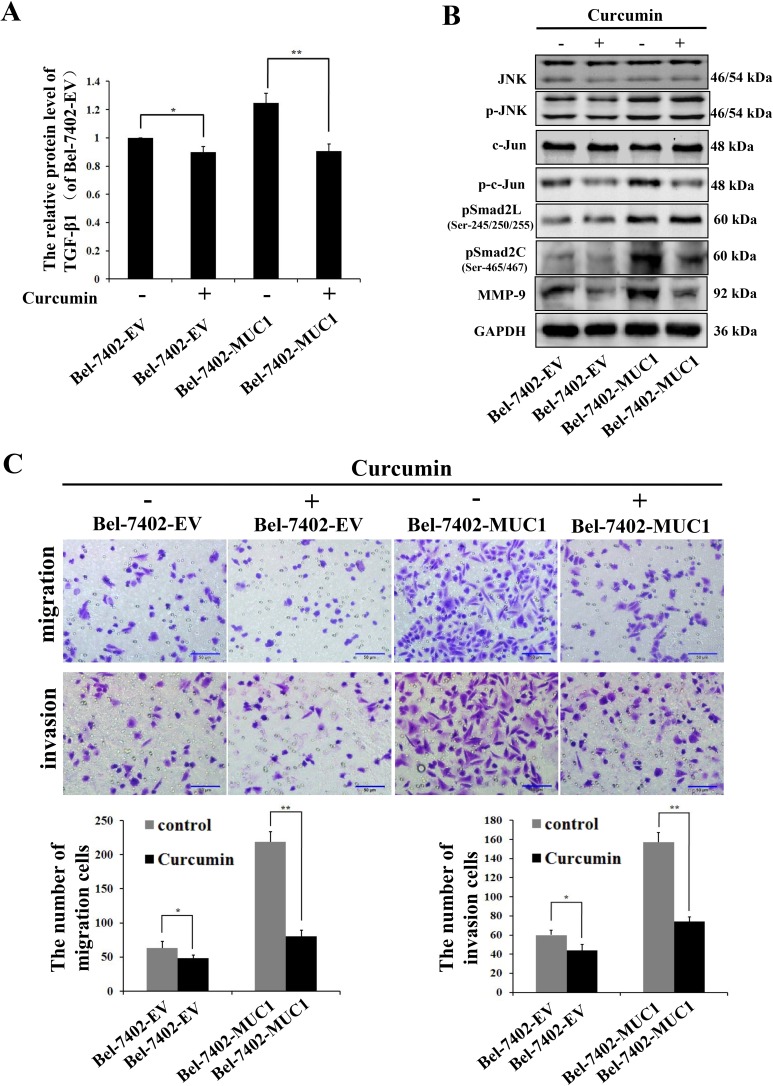
MUC1 promotes the migration and invasion of HCC cells through the JNK/AP-1/TGF-β signaling pathway **A.** The expression of TGF-β1 in Bel-7402-EV and Bel-7402-MUC1 cells when treated with or without Curcumin (20 μM) were assayed by ELISA. Bars represent the relative protein levels of TGF-β1 when compared to Bel-7402-EV (Curcumin−). The data are expressed as the mean ± SD of three independent experiments. **B.** JNK, p-JNK, c-Jun, p-c-Jun, pSmad2L (Ser-245/250/255), pSmad2C (Ser-465/467), and MMP-9 in Bel-7402-EV and Bel-7402-MUC1 cells when treated with or without Curcumin (20 μM) were analyzed by Western blotting; GAPDH was used as a loading control. **C.** The migration and invasion of Bel-7402-EV and Bel-7402-MUC1 cells when treated with or without Curcumin (20 μM) were detected by transwell migration and matrigel invasion assays, respectively. Migrated or invaded cells were counted in five random fields of each filter under a microscope (IX71; OLYMPUS) using a 200 × magnification. The scale bar indicates 50 μm. Bars represent the average number of migrated or invaded cells. *P < 0.05, **P < 0.01 compared with respective controls.

To further verify whether MUC1 promoted the migration and invasion of HCC cells through the JNK/AP-1/TGF-β signaling pathway, the JNK/AP1 pathway was blocked by Curcumin (20 μM), an inhibitor of AP-1. The results showed that the phosphorylation of Smad2C and the expression of TGF-β1 and MMP-9 were more significantly inhibited by Curcumin in Bel-7402-MUC1 cells compared with Bel-7402-EV cells, and Curcumin had no inhibitory effect on the phosphorylation of Smad2L (Figure 6A and 6B). Furthermore, the migration and invasion of Bel-7402-MUC1 cells were obviously suppressed by Curcumin compared with that of Bel-7402-EV cells (Figure 6C). The results further indicate that MUC1 promote the migration and invasion of HCC cells through the JNK/AP-1/TGF-β signaling pathway besides the JNK/Smad2L pathway.

## DISCUSSION

MUC1, as an oncogene, is involved in several signaling pathways to promote cell migration and invasion of various cancers [15-20, 35]. However, whether MUC1 could promote the migration and invasion of HCC cells remains largely unclear. In this study, to investigate the effect of MUC1 on the migration and invasion of HCC cells, we used two independent MUC1-knockdown clones (MR1-D4 and MR1-D9) and two MUC1-overexpressing cell lines (Bel-7402-MUC1 and Hep3B-MUC1) that were previously established [22, 23] and then performed wound-healing, transwell migration, and matrigel invasion assays. The results demonstrated that MUC1 promoted the migration and invasion of HCC cells. Our previous study found that MUC1 gene silencing inhibited the growth of SMMC-7721 cells in *vitro* and in *vivo* [22], and MUC1 shifted Smad3 signaling from a tumor-suppressive to an oncogenic function in MUC1-overexpressing HCC cells [34]. All these results suggest that MUC1 promotes the proliferation and progression of HCC.

TGF-β plays a tumor suppressive role in normal epithelia, but accelerates the progression through enhancing cell proliferation, migration, and invasion in cancer cells [24-27], and our recent study reveals that MUC1 induces autocrine TGF-β in HCC cells [23], which suggests that a novel mechanism for MUC1 may promote the migration and invasion of HCC cells via TGF-β signaling. Thus, the TGF-β level was detected by ELISA, and we found that MUC1 induced autocrine TGF-β1 in HCC cells, consistent with our previous result [23]. To investigate the effect of TGF-β secretion on HCC cell migration and invasion, Bel-7402-EV and/or Bel-7402-MUC1 cells were treated with or without different doses of exogenous TGF-β1, TGF-β1 siRNAs or TβRI inhibitor (SB431542). The results revealed that MUC1-induced autocrine TGF-β promoted the migration and invasion of HCC cells. There is abundant experimental evidence to support the excess production of TGF-β in various types of tumors [36, 37], however, the mechanism underlying the secretion of TGF-β by cancer cells is unclear. Recent studies have shown that oncogenic HER2 and H-Ras induced TGF-β secretion in mammary epithelial cells [36], in combining with our present results, suggesting that oncogenes may be the key switch for shifting TGF-β signaling from suppression to promotion in established cancers.

As described above, MUC1 upregulated the HCC cell migration and invasion and is closely correlated to MUC1-induced TGF-β, but the molecular mechanism remains largely unknown. JNKs are known as stress-activated protein kinases (SAPK) and belong to the mitogen-activated protein kinase (MAPK) superfamily. JNKs are generally thought to play different roles in inflammation, differentiation, and apoptosis. Accumulating evidence suggests that the JNK pathway is required for cell migration [38, 39]. Chen *et al.* showed that MUC1 could activate JNK1 by directly binding to it through the MUC1 cytoplasmic domain (MUC1-CD) and inhibit cisplatin-induced apoptosis in human colon cancer HCT116 cells [14]. A previous report showed that oncogenes, such as Ras and Her2, activated JNK/AP1 pathway to promote TGF-β secretion by mammary epithelial cancer cells [36]. Our results have also shown that MUC1 facilitates the autocrine TGF-β signaling via the JNK/AP-1 pathway in HCC [23]. Therefore, this leads to the hypothesis that MUC1-induced autocrine TGF-β upregulated the migration and invasion of HCC cells via the activation of JNK. In this study, we found that MUC1 enhances the activation of JNK and cell migration and invasion are almost completely inhibited in MUC1-overexpressing HCC cells when JNK is blocked by SP600125, suggesting that MUC1-induced the activation of JNK promotes the cell migration and invasion of HCC. Over the past decade, JNKs have increasingly been recognized as an attractive molecule target for the treatment of cancers [40] and recent studies have shown involvement of JNK activation in hepatic carcinogenesis [41-43], making JNK to be a therapeutic target of human HCC development and progression. All of our described results demonstrate that JNK, upstream of the TGF-β signaling, is a more attractive therapeutic target for HCC compared to TGF-β.

**Figure 7:**
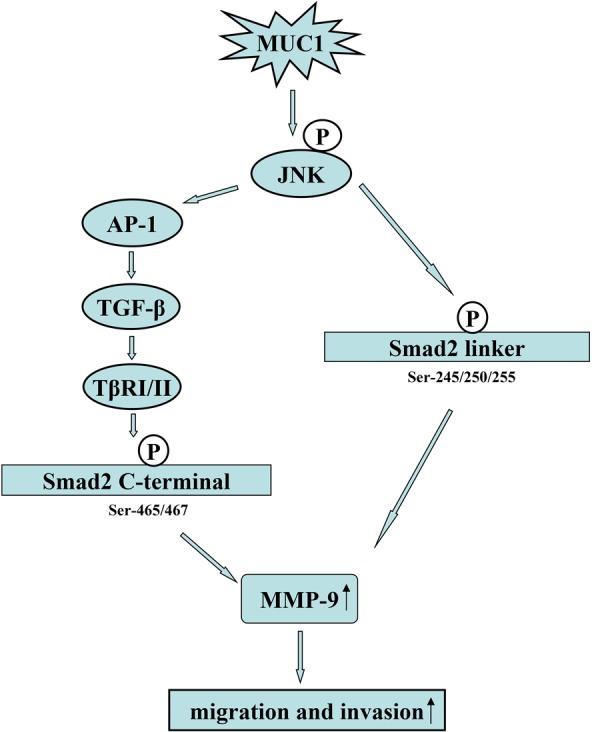
Proposed model for how MUC1 promotes the migration and invasion of HCC cells through activating JNK On the one hand, MUC1-mediated the activation of JNK enhances the autocrine TGF-β via AP-1, and then the autocrine TGF-β activates TβRI/II, further leading to the phosphorylation of Smad2C (Ser-465/467); on the other hand, MUC1-mediated the activation of JNK can directly phosphorylated Smad2L (Ser-245/250/255). Furthermore, the phosphorylated Smad2L and Smad2C promote the MMP-9-mediated cell migration and invasion of HCC.

Smads, central mediators converting TGF-β signaling from receptors to the nucleus, and TβRI can phosphorylate Smad2/3 in the C-terminal, which is a classical pathway. Studies by our group and other research groups have shown that phosphorylated Smad3 at the linker region promotes cancer cell proliferation [34, 43]. Francesco Dituri *et al.* showed that phosphorylated Smad2, but not phosphorylated Smad3, played a positive role in regulating TGF-β-induced migration [44]. Recently, Matsuzaka *et al.* also revealed that JNK can phosphorylate Smad2L and then the phosphorylation of Smad2L/C could promote HCC cell migration and invasion by upregulation of PAI-1, MMP-1, MMP-2 and MMP-9 [32, 33]. MMPs, a family of zinc-containing proteolytic enzymes, facilitate tumor invasion and metastasis [45], and an enhanced expression of MMP-9 has been shown to be correlated with the progression and invasion of HCC [46]. To investigate whether a molecular mechanism downstream of JNK is involved in the migration and invasion of HCC cells, the phosphorylation of Smad2L/C and the expression of its target genes were detected. In the present study, the results of qRT-PCR revealed that the mRNA level of MMP-9 but not those of PAI-1, MMP-1, or MMP-2 (data not shown) were consistent with the migration and invasion activities of both MUC1 gene silenced and MUC1-overexpressing HCC cells. We further found that MUC1 upregulated the phosphorylation of Smad2L/C and the expression of MMP-9 by Western blotting analysis. Especially, when Bel-7402-EV and Bel-7402-MUC1 cells were treated with or without different doses of exogenous TGF-β1, TGF-β1 siRNAs, JNK inhibitor SP600125 or TβRI inhibitor SB431542, we found that MUC1-induced JNK activation not only enhanced the phosphorylation of Smad2C through TGF-β/TβRI but also directly enhanced the phosphorylation of Smad2L, and then both of them collaborated to upregulate the MMP-9-mediated cell migration and invasion of HCC cells, which was consistent with the observations by Matsuzaki *et al.* [32, 33]. In addition, when the JNK/AP1 pathway was blocked by Curcumin, the results we obtained further confirmed this conclusion. These results suggest that MUC1 promotes the migration and invasion of HCC cells through the JNK/AP-1/TGF-β signaling pathway. As described in Figure 3 and 4, the migration and invasion of HCC cells were almost completely inhibited when JNK was blocked by SP600125, while blocking TGF-β signaling partly inhibited the migration and invasion of HCC cells, which also strongly suggests that JNK-mediated the phosphorylation of Smad2L and JNK/AP-1/TGF-β-mediated the phosphorylation of Smad2C in HCC cells are two important signaling arms required for maximizing MUC1-promoted HCC cell migration and invasion (Figure 7).

In summary, our study reveals that MUC1 promotes the migration and invasion of HCC cells via JNK-mediated the phosphorylation of Smad2L/C pathway, providing the theory basis for the target therapy of HCC. Although TGF-β signaling has been considered a therapeutic target in cancers, our present results demonstrate that MUC1 is an attractive target in liver cancer therapy.

## MATERIALS AND METHODS

### Cell lines and culture

The HCC cell lines SMMC-7721, Bel-7402 and Hep3B were purchased from the Cell Bank of the Shanghai Institute of Cell Biology, Chinese Academy of Sciences. Cells were cultured in Iscove's modified Dulbecco's medium (IMDM) supplemented with 100 U/ml penicillin, 100 μg/ml streptomycin, and 10% fetal bovine serum (FBS; Gibco-BRL, Carlsbad, CA, USA in an incubator at 37 °C and 5% CO2. The stable MUC1-knockdown cells (MR1-D4 and MR1-D9) and negative control cells (NC) of SMMC-7721 were generated as previously described [22] and maintained with 600 μg/ml G418 (Sigma). Two MUC1-overexpressing cell lines (named Bel-7402-MUC1 and Hep3B-MUC1) and two negative controls (named Bel-7402-EV and Hep3B-EV) were established in Bel-7402 and Hep3B cells, respectively, using Lentiviral Vector as previously described [23].

### Wound-healing assay

SMMC-7721, Bel-7402, and Hep3B cells (3 × 10^5^ cells/well) were seeded into 24-well plates at 80−90% confluency, and the cell monolayer was wounded with a 200-μl pipette. After washing with PBS for three times, 500 μl IMDM medium containing 1% FBS was added to the 24-well plates. The remaining cells were cultured in an incubator at 37 °C and 5% CO2. Cell migration was monitored under a microscope (IX71, OLYMPUS, JAPAN) at 100 × magnification at 0, 24 h, and 48 h. The wound areas were measured by Image-Pro Plus 6.0 software. The changes in migration were determined by comparing the difference in wound-healing areas within 48 h (*n* = 5).

### Transwell migration and matrigel invasion assays

Transwell migration and matrigel invasion assays were performed using a transwell membrane (8-μm pore size, 6.5-mm diameter; Corning Incorporated, Corning, NY, USA) in a 24-well plate according to the manufacturer's instructions. A matrigel matrix (1:5 Dilute, 50 μl/well, BD Biosciences) was coated in the transwell membrane (8-μm pore size, 6.5-mm diameter; Corning Incorporated, Corning, NY, USA) and used for the cell invasion assay. The lower chamber of the transwell plates was filled with 600 μl IMDM medium containing 10% FBS. HCC cells were detached from the tissue culture plates and resuspended in IMDM medium containing 1% FBS and then loaded to the upper side of the chamber (200 μl/well). For the migration assay, SMMC-7721 (1 × 10^5^ cells/well), Bel-7402 (5 × 10^4^ cells/well), and Hep3B (5 × 10^4^ cells/well) were used. For the invasion assay, SMMC-7721 (2 × 10^5^ cells/well), Bel-7402 (1 × 10^5^ cells/well), and Hep3B (1 × 10^5^ cells/well) were used. Notably, JNK inhibitor SP600125 (abcam), TβRI inhibitor SB431542 (abcam), activator protein 1 (AP-1) inhibitor Curcumin (abcam), and TGF-β1 (peprotech) were prepared with serum-free IMDM medium and then used to suspend the cells. The cells were placed in incubators at 37 °C for different time periods according to preliminary experiments. The filter inserts were then removed from the wells. Cells on the upper surface of the filter were removed using cotton swabs. Those on the lower surface were fixed with 4% paraformaldehyde in PBS, stained with 0.1% crystal violet and counted. Cells that migrated or invaded were counted in five random fields of each filter under a microscope (IX71, OLYMPUS, JAPAN) at 200 × magnification.

### Enzyme-linked immunosorbent assay (ELISA)

Cells were grown in complete medium and treated with or without JNK inhibitor (30 μM) and AP-1 inhibitor (20 μM) for 16 h and then incubated for 24 h in serum-free medium. Cell culture supernatants were assayed using a TGF-β1 ELISA kit (eBiosence) according to the manufacturer's instructions. The absorbance at 450-nm wavelength in each well was measured, and the concentration of TGF-β1 was calculated by comparison with the standard curve.

### Western blotting analysis

Western blotting analysis was performed as previously described [47]. The primary antibodies against MUC1 (CD227) (1:1000; NeoMarkers), GAPDH (1:10000), p-JNK (1:1000), JNK (1:1000), c-Jun (1:1000), p-c-Jun (1:1000), Phospho-Smad2 (Ser-465/467) (1:1000), and Phospho-Smad2 (Ser-245/250/255) (1:1000) were all purchased from Cell Signaling Technology, Inc. (Danvers, MA, USA).

### Quantitative real-time PCR (qRT-PCR)

The quantitative real-time PCR was performed according to standard protocols as previously described [22]. All primers used for qRT-PCR analysis were synthesized by Sangon Biotech, Shanghai, China. The primers used were as follows: MMP-9 forward: 5′-GAACCAATCTCACCGACAG-3′ and MMP-9 reverse 5′-GACTCTCCACGCATCTCT-3′; TGF-β1 forward: 5′-GCCCTGGACACCAACTATTG-3′ and TGF-β1 reverse 5′-CGTGTCCAGGCTCCAAATG-3′; and β-actin forward: 5′-AGTTGCGTTACACCCTTTC-3′ and β-actin reverse: 5′-CCTTCACCGTTCCAGTTT-3′.

### Interfering RNA (RNAi)

Two pairs of siRNA oligonucleotides corresponding to the target sequence for human TGF-β1: GGACTATCCACCTGCAAGA and GCAGAGTACACACAGCATA, respectively, were designed and synthesized by RiboBio (RiboBio Co. Ltd, Guangzhou, China). A negative-control siRNA (NC siRNA) (RiboBio) was used as medium control and a negative-control siRNA-Cy3 (RiboBio) was used to detect the transfection efficiency. Cells were cultured in IMDM containing 10% FBS without antibiotics, and were plated at 2 × 10^5^ cells per well in 12-well plates and grown to 30−50% confluent prior to transfection. The siRNAs were transfected into Bel-7402-EV and Bel-7402-MUC1 cells using Lipofectamine 2000 (Invitrogen). Cells were analyzed 24−72 h after transfection, and TGF-β1 mRNA expression was determined using qRT-PCR.

### Statistical analysis

Data are expressed as the mean ± SD. SPSS 21.0 software was used for analysis. All experiments were repeated at least three times. The statistical signiﬁcance of a difference between two groups was assessed using Student's t-tests, and *P* < 0.05 was considered to indicate a statistically significant result.
